# Skeletal muscles of hibernating black bears show minimal atrophy and phenotype shifting despite prolonged physical inactivity and starvation

**DOI:** 10.1371/journal.pone.0215489

**Published:** 2019-04-18

**Authors:** Mitsunori Miyazaki, Michito Shimozuru, Toshio Tsubota

**Affiliations:** 1 Department of Physical Therapy, School of Rehabilitation Sciences, Health Sciences University of Hokkaido, Hokkaido, Japan; 2 Laboratory of Wildlife Biology and Medicine, Graduate School of Veterinary Medicine, Hokkaido University, Hokkaido, Japan; University of Minnesota Medical Center, UNITED STATES

## Abstract

Hibernating mammals experience prolonged periods of torpor and starvation during winter for up to 5–7 months. Though physical inactivity and malnutrition generally lead to profound loss of muscle mass and metabolic dysfunction in humans, hibernating bears show limited muscle atrophy and can successfully maintain locomotive function. These physiological features in bears allow us to hypothesize that hibernating bears uniquely alter the regulation of protein and energy metabolism in skeletal muscle which then contributes to “muscle atrophy resistance” against continued physical inactivity. In this study, alteration of signaling pathways governing protein and energy metabolisms was examined in skeletal muscle of the Japanese black bear *(Ursus thibetanus japonicus)*. Sartorius muscle samples were collected from bear legs during late November (pre-hibernation) and early April (post-hibernation). Protein degradation pathways, through a ubiquitin-proteasome system (as assessed by increased expression of murf1 mRNA) and an autophagy-dependent system (as assessed by increased expression of atg7, beclin1, and map1lc3 mRNAs), were significantly activated in skeletal muscle following hibernation. In contrast, as indicated by a significant increase in S6K1 phosphorylation, an activation state of mTOR (mammalian/mechanistic target of rapamycin), which functions as a central regulator of protein synthesis, increased in post-hibernation samples. Gene expression of myostatin, a negative regulator of skeletal muscle mass, was significantly decreased post-hibernation. We also confirmed that the phenotype shifted toward slow-oxidative muscle and mitochondrial biogenesis. These observations suggest that protein and energy metabolism may be altered in skeletal muscle of hibernating bears, which then may contribute to limited loss of muscle mass and efficient energy utilization.

## Introduction

Skeletal muscle mass is generally determined by the net dynamic balance of protein synthesis and degradation [1]. Under catabolic conditions, muscle protein degradation is enhanced through ubiquitin-proteasome and autophagy-lysosome systems [2]. The muscle-specific E3 ubiquitin ligases atrogin1 and murf1 (muscle RING finger 1) are highly expressed in response to unloading/inactivity and contribute to protein ubiquitination and proteasome-dependent degradation [3, 4]. Autophagosome formation and lysosomal degradation of cytoplasmic components/organelles are also enhanced under catabolic conditions in skeletal muscle [5, 6]. In contrast, decreases in muscle protein synthesis have been observed in several animal and human models of disuse atrophy [7, 8]. Metabolic dysfunction is also induced in skeletal muscle following long-term disuse [9]. Consequently, prolonged periods of disuse lead to skeletal muscle atrophy/weakness and metabolic dysfunction, which then can cause impaired locomotive function and increased risk of morbidity/mortality.

Physiological characteristics during hibernation are highly variable between animal species. In general, small hibernators, such as ground squirrels, repeat torpor and arousal cycles every 1–2 weeks during hibernation periods. The thermal and energy metabolism are robustly down-regulated during torpor phase (body temperature: 3°C –5°C, heart rate: less than 10 bpm), whereas they return to the normothermic state (body temperature: 37°C, heart rate: 300–400 bpm) during short arousal phases. Eating, drinking, and excretion (defecation/urination) behaviors are observed during hibernation periods when the animals are awaking [10–12]. In contrast, larger hibernators, such as bears, experience no arousal (i.e., remain in a relatively shallow torpor) and do not eat, drink, urinate, or defecate during the entire period of hibernation even though they maintain thermal and energy metabolism at relatively high levels (i.e., body temperature is slightly decreased but maintained to 30°C –36°C) [13–15].

Although hibernating animals experience long-term inactivity and fasting during winter survival, they can successfully maintain their muscle mass and locomotive function until arousal in spring. Previous reports have indicated that skeletal muscle mass and strength are well preserved during hibernation. Fiber size in skeletal muscle was almost perfectly maintained during hibernation in small mammals (e.g., ground squirrels) [16, 17]. In the case of bears (e.g., American black bears or brown bears), protein content slightly decreased but loss of muscle mass and decline in contractile function were limited during hibernation [18–22]. These physiological features in hibernating animals allow us to hypothesize that the skeletal muscle of hibernators possesses a potential resistance to muscle atrophy during continued physical inactivity and malnutrition. In this study, alteration of signaling pathways that govern protein and energy metabolism was examined in skeletal muscle of the Japanese black bear.

## Materials and methods

### Antibodies

Mouse anti-dystrophin, developed by Morris, G.E. (DSHB Hybridoma Product MANDRA1(7A10)), was obtained from the Developmental Studies Hybridoma Bank, created by the NICHD of the NIH and maintained at The University of Iowa, Department of Biology, Iowa City, IA, USA 52242. Phospho-S6K1 (T389, Cat#: 9205) and phospho-S6K1 (T421/S424, Cat#: 9204) were obtained from Cell Signaling Technology (Danvers, MA, USA). S6K1 (Cat#: sc-230) was obtained from Santa Cruz Biotechnology (Santa Cruz, CA, USA). IRDye 680LT Goat anti-Rabbit IgG (Cat#: 926–68021) was from LI-COR Biosciences (Lincoln, NE, USA). Alexa Fluor 568-conjugated Goat anti-Mouse IgG (Cat#: A11031) was from Thermo Fisher Scientific (Rockford, IL, USA).

### Animal care and use

All experimental procedures and animal care performed in this study were conducted in accordance with institutional Guidelines for Animal Care and Use, as approved by the Animal Care and Use Committee of the Graduate School of Veterinary Medicine, Hokkaido University (Permit Number: 9124). A total of six non-pregnant female Japanese black bears *(Ursus thibetanus japonicus)*, between 5 to 15 years of age, kept in Ani Mataginosato Bear Park (Akita Prefecture, Japan, N40° E140.4°) were used in this study. All animal care and handling procedures were followed as previously described [14, 23]. Briefly, animals were fed with dried corn (360 kcal/100 g, approximately 1.5 kg/head) combined with fruits and vegetables as supplements once a day at 16:00 h during the active period (i.e., from late April to late November). During the two weeks before or after the fasting period (i.e., late November/early December to early/mid-April) as a transition phase to/from torpor status, the amount of feeding was reduced to one-third (0.5 kg corn meal/head) compared to the active period. All animals were kept isolated in the indoor dark rooms for denning and had no access to the food during the torpor period. Access to drinking water was allowed ad libitum throughout the year.

### Muscle sample collection

Skeletal muscle samples (sartorius muscle) were collected from bear legs during both pre-hibernation state (i.e., late November, just prior to the beginning of food deprivation) and post-hibernation state (i.e., early April, one week following the onset of re-feeding). Immediate post-hibernation period (approximately 3 weeks following emerged from denning) was known as “walking hibernation” [15]. During this period, the biochemical stage of hibernation was likely persisted in bears, even though physical activity and food/water intake were minimal. Due to the limitation of animal availability, different individual bears were used at each time point (N = 3 in each group). Animals were anesthetized with an intramuscular administration of 3.0 mg/kg zolazepam HCL and tiletamine HCL cocktail and 40 μg/kg medetomidine HCL using a blow dart shot. After immobilization, small pieces of sartorius muscle were excised directly and quickly frozen in liquid nitrogen. Feeding was restricted overnight (about 15–16 h) until the anesthesia and sample collection surgery were completed the next morning. Following the sample collection surgery, meloxicam (subcutaneously at 0.2 mg/kg for analgesia) and atipamezole HCl (intramuscularly at 200 μg/kg as an antagonist to medetomidine HCL) were administered to aid recovery.

### Histochemistry/immunohistochemistry

Muscle samples for histochemical or immunohistochemical analyses were frozen in liquid nitrogen–cooled isopentane. Cross sections (8 μm) were cut in a cryostat (Leica CM 1860, Leica Biosystems, Eisfeld, Germany) and stored at −80°C until analysis. For hematoxylin/eosin (HE) staining, sections were fixed in 4% paraformaldehyde (PFA), stained with Mayer's hematoxylin solution for 5 min and then with eosin solution for 5 min. For the NADH-tetrazolium reductase (NADH-TR) staining, sections were incubated for 30 min at 37°C in a reaction mixture containing 1.5 mM NADH and 1.5 mM nitrotetrazolium blue in 200 mM Tris (pH 7.4). For the immunohistochemical analysis, sections were fixed in 4% PFA, permeabilized with 0.1% Triton X-100, and blocked with 1% bovine serum albumin. Mouse anti-dystrophin and Alexa Fluor 568-conjugated goat anti-mouse IgG antibodies were used for detecting dystrophin localization. Sections were mounted with Vectashield mounting medium for microscopic observation. All images were captured using the OLYMPUS IX73 system and cellSens imaging software (OLYMPUS, Tokyo, Japan). Cross-sectional areas (CSA) of muscle fibers were measured using dystrophin-stained 20X magnification images and WinROOF image analysis software (MITANI corporation, Tokyo, Japan).

### RNA isolation and real-time PCR

Total RNA was prepared using the TRIzol Reagent (Thermo Fisher Scientific, Rockford, IL, USA) according to the manufacturer's directions. RNA samples were treated with TURBO DNA-free (Thermo Fisher Scientific, Rockford, IL, USA) to remove genomic DNA contamination. Isolated RNA was quantified using spectrophotometry (λ = 260 nm). First-strand cDNA synthesis from total RNA was performed using the PrimeScript RT Reagent Kit. SYBR Premix Ex Taq II and TaKaRa Thermal Cycler Dice Real Time System TP850 (Takara Bio, Shiga, Japan) were used for PCR amplification and quantification of each studied gene. Primer sequences were designed based on partial sequencing of each gene obtained from the Japanese black bear and/or the American black bear [24] using Primer3 software. Expression levels of each studied gene were determined by the 2^–ΔΔCT^ method with referencing ribosomal protein L26 as an internal control.

### Protein extraction and western blotting

For protein extraction, tissue samples were lysed in ice-cold RIPA buffer (1% NP-40, 0.5% sodium deoxycholate, 0.1% SDS, 50 mM NaCl, 20 mM Tris–HCl [pH, 7.6], 1 mM PMSF, 5 mM benzamidine, 1 mM EDTA, 5 mM N-ethylmaleimide, 50 mM NaF, 25 mM B-glycerophosphate, 1 mM sodium orthovanadate, and 1X protease inhibitor cocktail [Nacalai Tesque, Kyoto, Japan]). Lysed samples were then centrifuged at 16,000 × g for 10 min at 4°C, and supernatants were collected for analysis. Protein concentration was determined using the BCA Protein Assay Kit (Thermo Fisher Scientific, Rockford, IL, USA). Protein samples were separated using a precast polyacrylamide gel system (e-PAGEL; ATTO, Tokyo, Japan) and transferred to PVDF membranes. Membranes were then blocked in Odyssey Blocking Buffer and incubated with dilutions of each primary antibody. IRDye 680LT goat anti-rabbit IgG was used as secondary antibody. Bound antibody complexes were scanned and quantified using the Odyssey CLx Imaging System operated with Image Studio Version 3.1 software (LI-COR Biosciences, Lincoln, NE, USA).

### Statistical analysis

All results are reported as means ± standard error. Statistical differences between pre-hibernation (November) and post-hibernation (April) were determined by the Student's t-test. For all comparisons, the level of statistical significance was set at p < 0.05.

## Results and discussion

The “Use It or Lose It” phenomenon is a well-accepted physiological principle for skeletal muscles. Skeletal muscle is highly plastic in response to functional demands, such that a reduced level of contractile activity (i.e., disuse) typically leads to skeletal muscle loss and metabolic dysfunction in many animal species, including humans. However, hibernating animals are likely better described under the principle “Limited Use, but Limited Loss” phenomenon, in that there is a potential resistance to muscle atrophy during continued disuse conditions. In the study presented herein, we analyzed alteration of signaling pathways regulating protein and energy metabolism in skeletal muscle of hibernating bears.

### Alteration of muscle fiber cross-sectional areas: Pre-hibernation vs. post-hibernation

**Fig 1:**
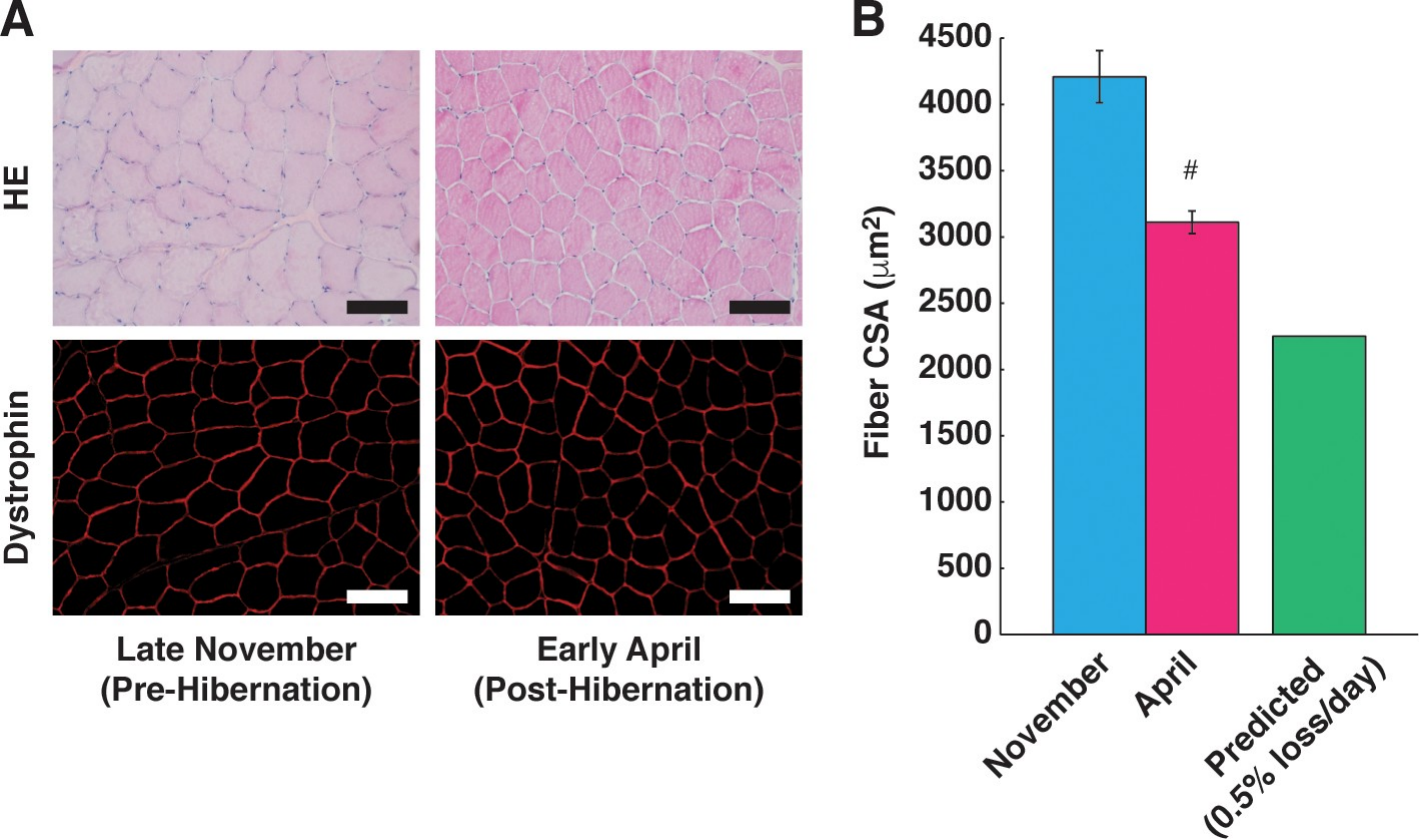
Alteration of Muscle Fiber Cross-Sectional Areas: Pre-Hibernation vs. Post-Hibernation. Fiber cross-sectional areas (CSAs) were significantly decreased following hibernation. The sartorius muscle was collected from bear legs both at pre-hibernation (late November) and post-hibernation (early April). Due to the limitation of animal availability, different individual bears were used at each time point (N = 3 in each group). (A) Typical images of cross sections with Hematoxylin-Eosin staining and immunohistochemistry (dystrophin localization) are shown. The scale bar shows 100 μm. (B) The mean size of the fibers was obtained for each individual sample followed by the calculation of group data. Data are expressed as mean ± standard error. Significant differences: #, p < 0.05.

HE staining revealed no seasonal differences in the gross morphology of muscle fibers in bears between pre-hibernation (late November) and post-hibernation (early April). We originally speculated that there is a very small reduction of fiber size in bear skeletal muscle following hibernation. In practice, however, muscle fiber size following hibernation decreased significantly (pre-hibernation, 4207.0 ± 194.6 μm^2^; post-hibernation, 3111.6 ± 87.2 μm^2^; 26% decrease). This result was consistent with previous reports demonstrating that force generation capacity [21] or protein concentration [18] in bear skeletal muscle was significantly decreased following hibernation. These observations clearly indicate that muscle atrophy is essentially induced following hibernation in bears. Although skeletal muscle atrophy had happened, this decrease in muscle fiber size following hibernation in bears seems to be limited. Daily loss of muscle size or muscle protein content was predicted at about 0.5%–1.0% under disuse conditions in human subjects [25, 26]. If loss of muscle fiber size were to occur at 0.5%, an approximate 53% decrease would be expected after five months (150 days) of hibernation. Therefore, while we observed a significant decrease in muscle fiber size following hibernation, it is possible that over 27% of predicted muscle loss was protected during hibernation (Fig 1).

### Regulation of muscle protein catabolism

**Fig 2:**
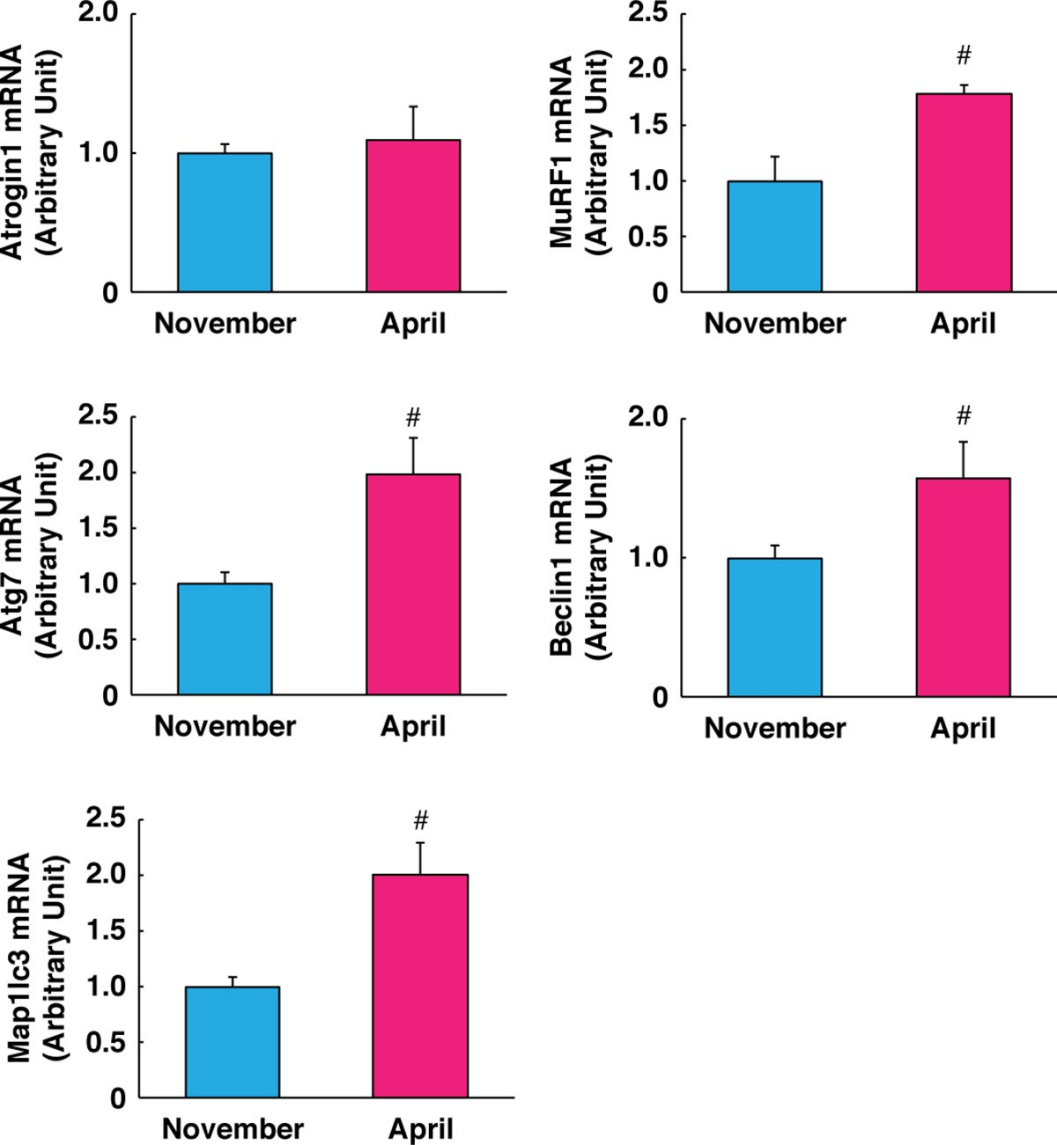
Gene expression of protein breakdown pathways. Gene expression levels of the ubiquitin-proteasome system (*atrogin1* and *murf1*) and autophagy-lysosome system (*atg7*, *beclin1*, and *map1lc3*) were quantified by real-time PCR. RPL26 was used as an internal control for the 2^–ΔΔCT^ method. N = 3 in each group. Data are expressed as mean ± standard error. Significant differences: #, p < 0.05.

Next, we evaluated the activation status of protein breakdown regulation. In skeletal muscle, there are two major pathways of protein breakdown which are activated during muscle atrophy. The ubiquitin-proteasome pathway is generally activated under disuse conditions in skeletal muscle. Autophagy-dependent protein breakdown is activated under poor nutrition states such as long-term fasting or malnutrition. Although we could not detect significant alteration in *atrogin1* expression, transcript level of *murf1*, a muscle-specific E3 ubiquitin ligase, was significantly increased. In addition, expression levels of autophagy-related genes, including *atg7*, *beclin1*, and microtubule-associated protein 1 light chain 3 (*lc3*), were significantly up-regulated following hibernation. These results suggest that protein degradation pathways through ubiquitin-proteasome-dependent and autophagy-dependent systems were both likely activated in skeletal muscle in response to the long-term disuse and malnutrition environment of hibernation (Fig 2). In contrast, a previous report investigating muscle protein metabolism directly by using radio-isotope tracers showed that protein synthesis and breakdown were both lowered during winter-denning period in American black bears [22]. The reason for this contradicted observation is still unknown. However, increased expression of autophagy-related genes in skeletal muscle following hibernation revealed the possibility that autophagy-dependent proteolysis could contribute to amino acid production as an alternative energy resource in response to long-term fasting. One other possibility could be related to the seasonal effects of sample collection periods. In this study, the muscle samples were collected at the immediate post-hibernation state (i.e., 1 week following the onset of re-feeding), as opposed to collection during denning periods as in previous report [22]. Although the biochemical stage of hibernation persisted in bears during the immediate post-hibernation period (known as “walking hibernation”) [15], protein metabolism in skeletal muscle could have been potentially affected by arousal and physical activity status.

### Regulation of muscle protein anabolism

**Fig 3:**
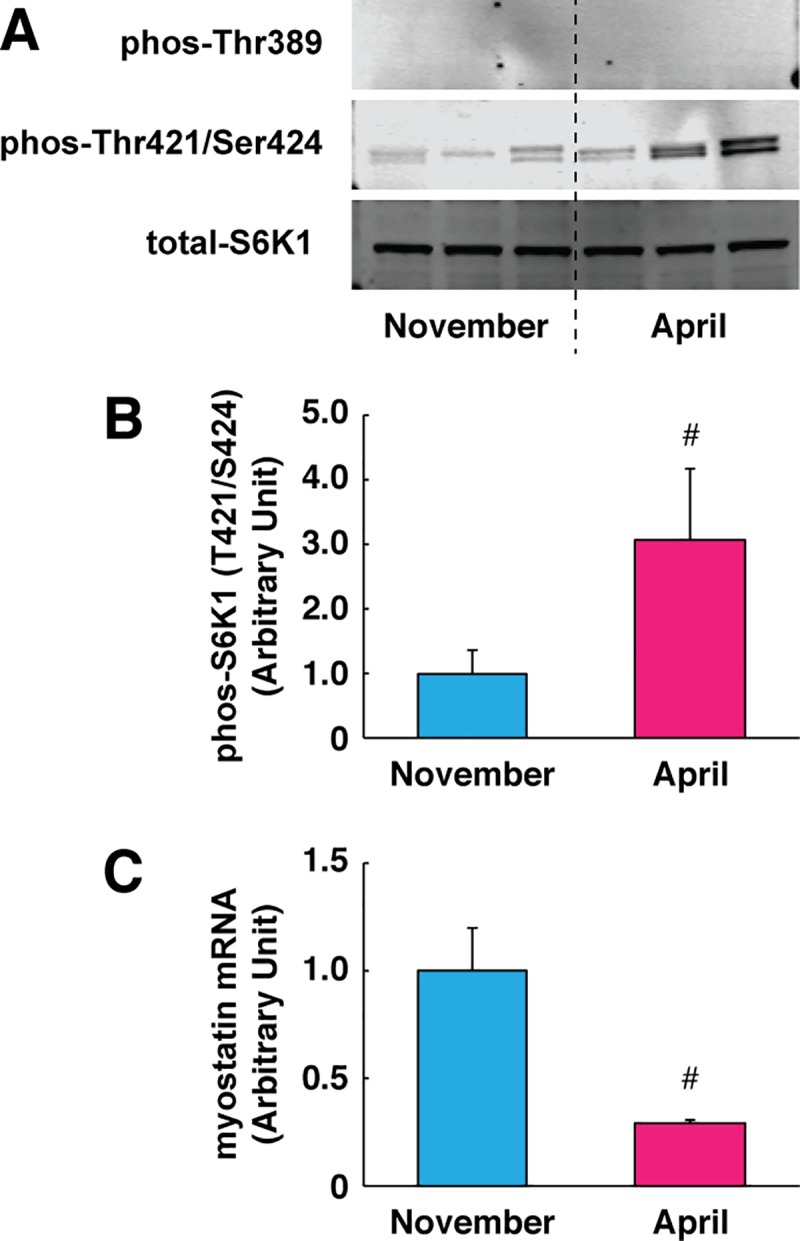
Enhanced mTORC1 signaling and suppression of myostatin expression following hibernation. (A) Representative images of Western blotting for phosphorylated (Thr389 and Thr421/Ser424) and total expression of S6K1. (B) Phosphorylation status of S6K1 at Thr421/Ser424 sites were quantified. (C) Gene expression levels of myostatin were determined by real-time PCR. RPL26 was used as an internal control for the 2^–ΔΔCT^ method. N = 3 in each group. Data are expressed as mean ± standard error. Significant differences: #, p < 0.05.

Together with the changes in muscle fiber size, the activation status of the mechanistic target of rapamycin (mTOR)-dependent signaling was examined in skeletal muscle. The mTOR complex 1 (mTORC1) has been suggested as a central regulator of protein synthesis through modulating translational efficiency from mRNA to amino acid, which could lead to increased activity of mTORC1 signaling resulting in enhanced protein synthesis. [1]. Interestingly, S6K1 phosphorylation that shows functional activity of mTORC1-dependent signaling was significantly increased following hibernation. We also observed that expression levels of myostatin mRNA was significantly reduced following hibernation. Myostatin is a negative regulator of skeletal muscle growth that reduces mTORC1 activity via Smad2/3 signaling [27]. Previous reports have indicated that skeletal muscle with decreased levels of myostatin show bulky musculature phenotypes in many animal species, including cattle, dogs, and humans [28, 29]. In bear skeletal muscle, myostatin mRNA expression was significantly down-regulated following hibernation (Fig 3). Measurements of the absolute rate of protein synthesis were not collected in this study. However, activation of mTORC1-dependent signaling and suppression of myostatin expression suggests that the growth response of bear skeletal muscle may be enhanced following hibernation. Conversely, previous reports have pointed out that protein synthesis rates in skeletal muscle were essentially diminished during hibernation compared with that of summer periods in American black bears [22]. We postulate that increased molecular responses (i.e., mTOR activation and myostatin down regulation), which can contribute to skeletal muscle growth, likely counteract a corresponding enhancement of protein degradation, which can then lead to a net balance of protein metabolism and prevention of excessive muscle loss. Other potential explanations could be related to the effects of nutrient availability and the energy status of muscle cells, as the post-hibernation muscle samples were collected at one week following the onset of re-feeding. Although the acute effect was minimized (i.e., feeding was restricted overnight until sample collection), potential amino acid availability in muscle cells could possibly reflect the nutrient-induced activation of the mTORC1 pathway.

### Phenotype shifting and mitochondrial biogenesis are enhanced in skeletal muscle following hibernation

**Fig 4:**
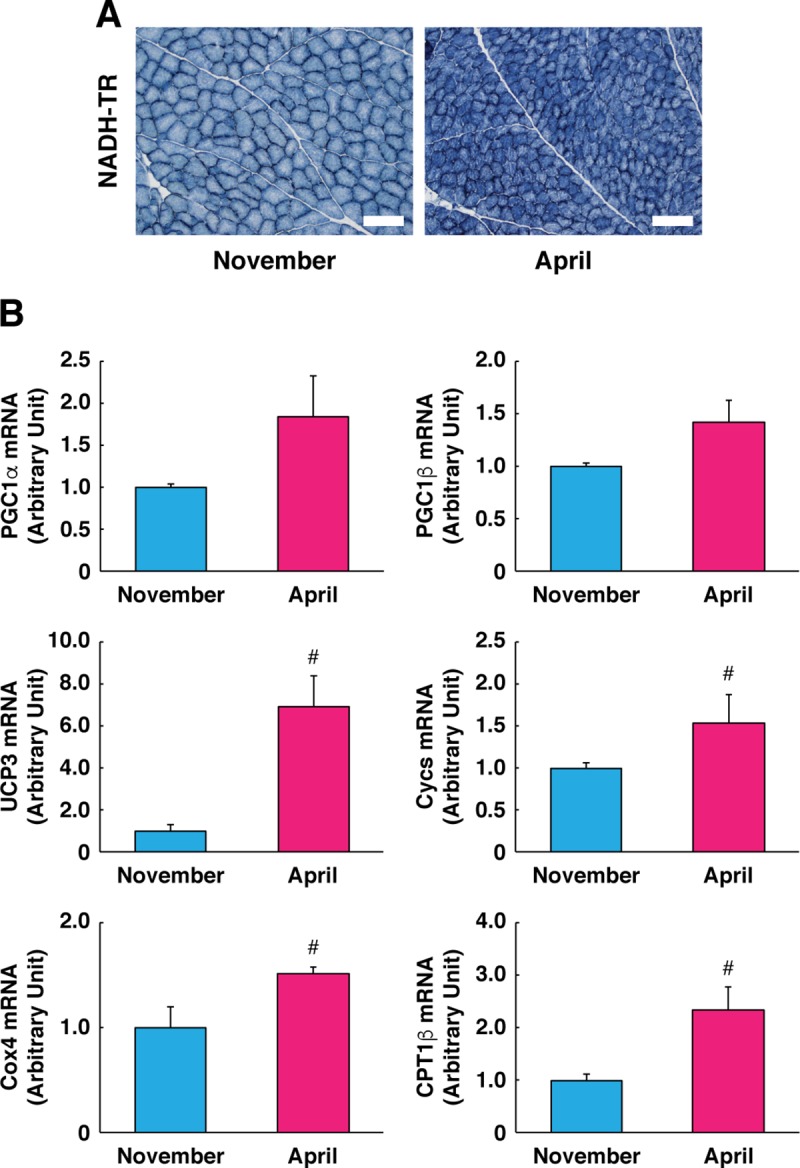
Expression of mitochondria-related genes following hibernation. (A) Typical images of cross sections with NADH-tetrazolium reductase (NADH-TR) staining are shown. The scale bar shows 200 μm. (B) Expression levels of mitochondria-related gene transcripts (*pgc1a*, *pgc1b*, *ucp3*, *cycs*, *cox4*, and *cpt1b*) were quantified by real-time RT-PCR. RPL26 was used as an internal control for the 2^–ΔΔCT^ method. N = 3 in each group. Data are expressed as mean ± standard error. Significant differences: #, p < 0.05.

We also examined the muscle fiber phenotypes in bear skeletal muscle. Long-term disuse generally results in a profound shifting from slow-oxidative to fast-glycolytic muscle fiber phenotypes [30]. However, histochemistry analyses demonstrated increased staining intensity of the mitochondrial enzyme NADH following hibernation. To confirm that the fiber type shifted toward the more oxidative/mitochondrial phenotype following hibernation, expression levels of mitochondrial genes in skeletal muscle were examined. Although the master regulators of mitochondrial biogenesis *peroxisome proliferative activated receptor*, *gamma*, *coactivator 1 alpha* (*pgc1a*) and *pgc1b* were not altered, expression levels of mitochondria-related genes were significantly up-regulated following hibernation, including the mitochondrial uncoupling protein *ucp3*, regulators of electron transport and complex formation (*cytochrome c*: *cycs* and *cytochrome c oxidase subunit 4*: *cox4*), and a regulator of fatty acid beta-oxidation (*carnitine palmitoyltransferase 1b*: *cpt1b*). These data support the idea that mitochondrial biogenesis had occurred following hibernation in bear skeletal muscle (Fig 4). This observation was also consistent with previous reports in other hibernating mammals showing that skeletal muscle fiber type shifted from the fast/glycolytic to slow-oxidative phenotype during hibernation [31–33]. Although muscle contractile activity was essentially minimal during hibernation, previous reports have indicated that peaks in average electromyogram amplitude were observed when shivering bursts occurred [13, 34]. We postulate that increased mitochondrial biogenesis in skeletal muscle could contribute to more efficient utilization of nutrients (e.g., fatty acids and/or glucose) as energy resources for muscle contraction during winter survival, particularly for shivering-induced thermogenesis through mitochondrial oxidative phosphorylation. Up-regulation of UCP3 mRNA also explains that increased mitochondrial biogenesis in skeletal muscle may contribute toward more efficient thermogenesis during hibernation [35, 36]. An alternative explanation for the observed increases in mitochondria-related genes in skeletal muscle may be attributed to the physiological alterations that accompany hibernation rather than an enhanced number/content of mitochondria. The fasted state could up-regulate UCP3 expression as a mediator of fatty acid metabolism in skeletal muscle [35]. Expression of CPT1, a regulating enzyme for fatty acid beta-oxidation, was also increased in skeletal muscle when the animals were exposed to cold ambient temperature [37]. Therefore, it will be important in future studies to evaluate whether enhanced mitochondrial biogenesis is induced and whether this contributes to improved mitochondrial respiratory function in skeletal muscle during hibernation.

Common disadvantages of studies using non-model organisms include limited information and research resources, including availability of sequenced genomes and mutants, and a lack of gene or protein expression profiles. Recent advances in sequencing technology offer unprecedented opportunities for research in non-model organisms. In hibernating animals, proteomic and transcriptomic analyses in skeletal muscle have been extensively explored with ground squirrels, mammals that exhibit typical physiological extremes in small hibernators [32, 33, 38–41]. Comprehensive works by Dr. Andrews’ lab, which investigated gene expression profiles in skeletal muscle of thirteen-lined ground squirrels, have identified seasonal alteration of differentially-expressed genes that may contribute to the regulation of muscle contractile function, protein catabolism, and energy homeostasis throughout the circannual cycle [32, 38–40]. The other study using proteomic analysis also indicated that physiological transitions for the hibernator skeletal muscle, such as changes in metabolic preference and fast-to-slow fiber type shifting have been induced [33]. Although availability of detailed omics datasets of larger hibernators is still very limited, some studies have attempted to identify the potential adaptive mechanisms in skeletal muscle during hibernation. A gene expression screening using cDNA microarray specifically developed for the American black bear identified 12 genes, mostly involved in protein biosynthesis, that were elevated in skeletal muscle during hibernation compared with summer active periods [24]. Elevated expression of genes involved in protein biosynthesis and ribosome biogenesis during hibernation was also observed in skeletal muscle of both small (arctic ground squirrels) and large (American black bear) hibernators [41]. These previous reports, along with our observations in this study, suggest that translational regulation of muscle protein possibly contributes to a common mechanism for “muscle atrophy resistance” in hibernating animals.

## Conclusion

**Fig 5:**
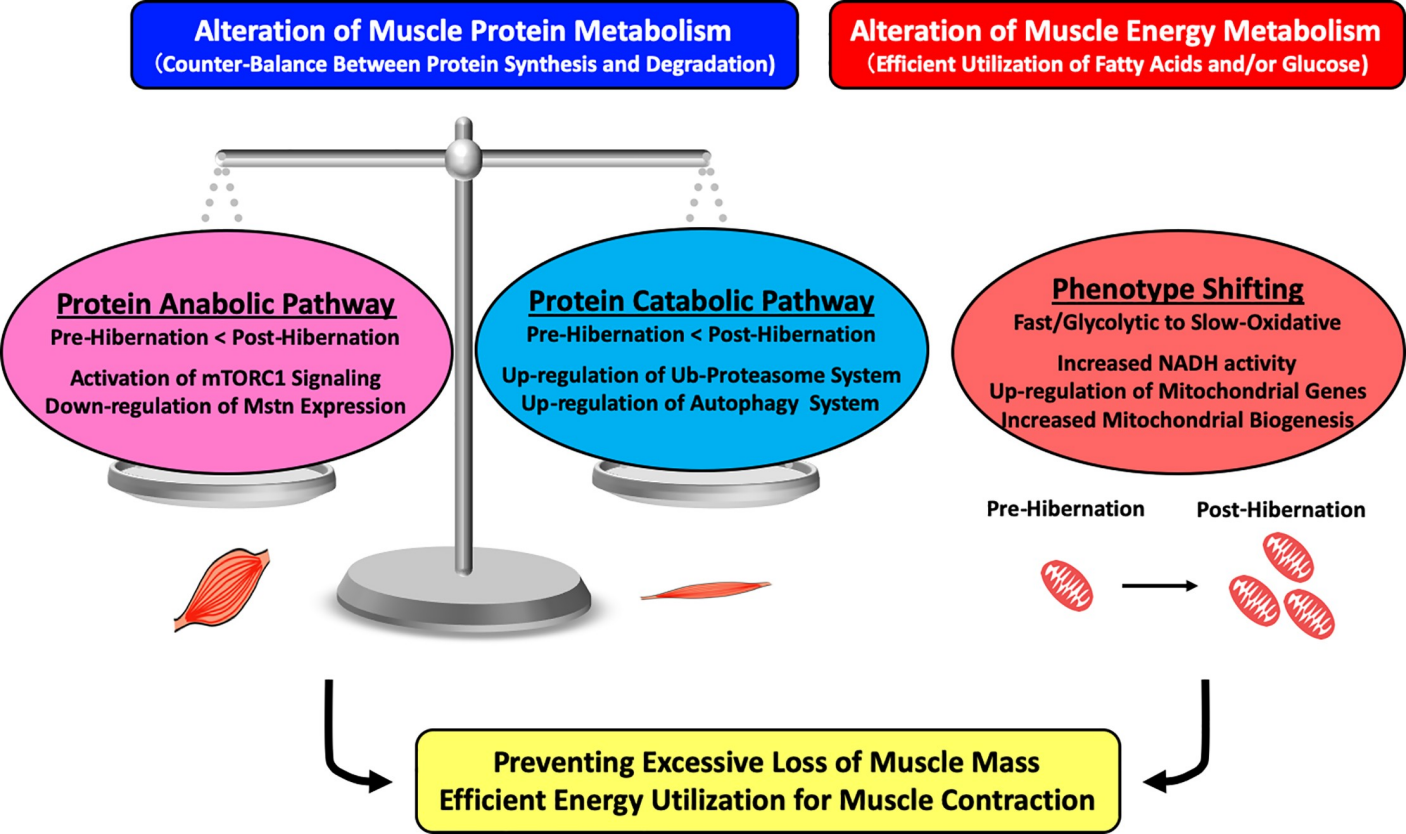
Hypothetical schema of skeletal muscle adaptation in hibernating bear. Protein anabolism in skeletal muscle of hibernating bears is potentially activated by mTORC1 activation and down regulation of myostatin expression for counteracting to the corresponding enhancement of protein degradation, which can then lead to a net balance of protein metabolism and prevention of excessive muscle loss. Muscle phenotypes shifting toward slow-oxidative fiber and mitochondrial biogenesis are also induced and likely contribute to the more efficient utilization of energy resources for muscle contraction during winter survival.

According to our results, muscle protein anabolism was potentially altered by mTOR activation and down regulation of myostatin expression following hibernation. This anabolic response of bear skeletal muscle may be a counteraction to enhanced muscle catabolism (i.e., increased ubiquitin-proteasome-dependent and autophagy-dependent systems) for preventing excessive loss of muscle mass following hibernation. We also observed muscle phenotypes shifting toward oxidative fiber and mitochondrial biogenesis. These alterations of energy metabolisms in skeletal muscle may contribute to the more efficient utilization of fatty acids and/or glucose as energy resources for muscle contraction during winter survival (Fig 5). Overall, these physiological adaptations in bear skeletal muscle could contribute to muscle atrophy/weakness resistance against long-term disuse and malnutrition during hibernation.
